# Give Goods or Give Money? The Influence of Cause-Related Marketing Approach on Consumers’ Purchase Intention

**DOI:** 10.3389/fpsyg.2020.533445

**Published:** 2021-01-22

**Authors:** Shenghong Ye, Yunxiao Liu, Suizi Gu, Haiquan Chen

**Affiliations:** School of Management, Jinan University, Guangzhou, China

**Keywords:** corporate social responsibility, cause-related marketing approach, product type, perceived helpfulness, information statement order

## Abstract

With the progress of social and improvement of public awareness, the demand for enterprises to participate in a social welfare cause is increasing. A company can directly support corporate social responsibility issues through cause-related marketing (CRM) approaches, for example, by donating part of the proceeds from product sales (i.e., buy-one give-money or BOGM) or simply by donating their products (i.e., buy-one give-one or BOGO). Previous research has only discussed the impact of one of these CRM approaches on customers in one study. This research compared the effect of these two approaches on the purchase intention of consumers. Experiment 1 demonstrated that, for practical products, the purchase intention of BOGO (vs. BOGM) was higher, while for hedonic products, the purchase intention of BOGM (vs. BOGO) was higher. More importantly, we found a potential mechanism – perceived helpfulness – that drives our main effect. Experiment 2 revealed that the different statement order of charity information and product information in advertising can moderate the main effect. The research also provides several implications and insight into how companies can make donations while winning more customers’ willingness to pay, thus encouraging more companies to fulfill their corporate social responsibility.

## Introduction

With the spread of concepts such as economic globalization, sustainable development, and harmonious society, the demands on social responsibility are increasing, which makes more and more firms invest their resources in solving social problems (e.g., environmental pollution, poverty, disease, and children’s education) and begin to make charitable donations through cause-related marketing (CRM) approach (Choi et al., 2018). Most executives noted that corporate social responsibility (CSR) measures could help them improve their long-term performance and achieve sustainable development. Half of the managers believed that long-term commitment to socially responsible behavior contributes to an excellent enterprise image (Bonini et al., 2010). It shows that companies can not only fulfill their social responsibilities through CRM but also obtain consumer support. Unlike other marketing campaigns, the most important feature of CRM is the commitment of companies to donate part of the sales from consumer purchases to charity (Koschate-Fischer et al., 2012). This is a form of marketing that realizes the interests and goals of consumers, charities, and businesses alike (Varadarajan and Menon, 1988).

The company’s marketing efforts usually focus on developing successful strategies to encourage consumers to participate in the brand and ultimately achieve marketing goals (Keller and Kotler, 2012). As an originally short-term promotional strategy, CRM has helped companies achieve their goals, and it has evolved into a popular and successful strategy nowadays. However, when an enterprise wants to implement CSR strategies, the decision of the enterprise needs to consider not only which social problems to contribute to and how much to donate (Yoo et al., 2018) but also how to contribute (e.g., cash, products, and employee volunteerism; Hildebrand et al., 2017). At present, there are two ways to realize social responsibility through CRM approach, buy-one give-one or buy-one give-money. For example, when a consumer purchases a pair of shoes from TOMS, TOMS would donate a pair of shoes to children in Africa or another place where children need shoes. The example of buy-one give-money is Taobao’s public welfare plan called “Gong Yi Bao Bei.” Specifically, when consumers buy any product with a charity label of “Gong Yi Bao Bei,” the charity will receive as donation a certain amount of money. Both approaches of CRM are often used by companies; the aim of this study is to find out which one can make consumers have a higher purchase intention.

With the application of cause marketing strategy, academic research of CRM has gained momentum. Previous empirical research on CRM has mainly focused on two aspects, one was from the enterprise perspective and the other on the influence of CRM on consumer attitudes, behavior, and intentions (Varadarajan and Menon, 1988). In a recent study, Lafferty et al. (2016) divided CRM studies into three areas: firm and cause (Chen et al., 2013; Boenigk and Schuchardt, 2014; Westberg and Pope, 2014; He et al., 2016; Kulow and Kramer, 2016), consumer (Grau and Folse, 2007; Kim and Johnson, 2013; Myers and Kwon, 2013), and CRM strategy and execution (Chang and Cheng, 2015; Coleman and Peasley, 2015; Hagtvedt and Patrick, 2016; Kulow and Kramer, 2016).

As for the current research, we try to discuss the effect of different CRM approaches on consumer purchase intention. Previous researches of cause-related marketing strategy have addressed the impact of different product types on CRM effects (Strahilevitz and Myers, 1998; Strahilevitz, 1999), but the impact of the interaction between CRM approaches and product types on CRM is still unclear. In addition, although there have been studies on the impact of the information framework (positive information framework vs. negative information framework) on CRM (Grau and Folse, 2007), few studies considered how information statement order affects the effectiveness of CRM. How does the interaction of CRM approach and product type affect consumer purchase intention? In some cases, would some kind of information statement order be more persuasive? This paper attempts to explore these issues.

## Literature Review and Hypotheses Development

### The Interaction Between the CRM Approach and Product Type

Cause-related marketing is a practical and popular form of CSR (Kotler and Lee, 2008) and is the process of formulating and implementing marketing activities, which is characterized by the willingness of firms to contribute a specified amount to a cause when customers engage in activities and earn sales revenue (Varadarajan and Menon, 1988). Charitable giving is defined as the donation of funds to an organization that benefits others beyond family (Bekkers and Wiepking, 2010). The researchers have extensively discussed the impact of CRM on consumer response (see Table 1).

**Table 1:** The effect of cause-related marketing (CRM) on consumer response.

Reference	Outcome variables	Effects of CRM on outcomes
Varadarajan and Menon (1988)	Purchase behavior	CRM will positively influence consumers’ initial and continuous purchase intention
Ross et al. (1992)	Product evaluation	Consumers had better evaluation and higher purchase preference for products with CRM and were willing to convert their current brands into corporate brands with a higher level of social responsibility
Sánchez (2000)	Advertising effectiveness	Charity marketing helped enterprises to establish a good image and brand recognition and had a better advertising effect
Chaney and Dolli (2001)	Actual purchase behavior	Most consumers would show their support for the CRM activities through actual purchase behavior
Kalligeros (2005)	Emotional connections and purchase behaviors	CRM could create emotional connections between consumers and products which could further affect consumers’ purchase behaviors
Lee Thomas et al. (2011)	Consumers’ attitudes and brand credibility	CRM made companies benefit from improvement of consumers’ attitudes and credibility toward brand
Moosmayer Dirk and Fuljahn (2013)	Evaluation of the firm	When consumers believed that the CRM campaign was based on altruistic motives, their evaluations of the firm would be improved significantly

The first CRM approach we focused on in this research, known as buy-one give-one (briefly described as BOGO subsequently), can be explained simply as a way for a company to donate the same product or a product of the same category to charity after a consumer purchased a product from the enterprise. As for buy-one give-money (briefly described as BOGM), it means that the company will donate money directly to the charity after a consumer purchased a product. Previous study has shown that the larger the donation magnitude, the stronger the consumer’s perception and purchase intention toward charitable marketing activities (Human and Terblanche, 2012; Yoo et al., 2018). In this study, we controlled BOGM contributions of the same size as the value of the product or a product of the same category in order to avoid the impact of donation magnitude on consumer’s purchase intention.

Scholars classify products into practical products and hedonic products according to their different properties. The former is more target-oriented and mainly used to meet the basic needs of consumers in specific functions, which are generally necessary for daily life. The latter is more oriented toward experiencing pleasure, which is mainly used to satisfy pleasure and for the enjoyment of the senses and spirits of a consumer who seeks an immediate emotional response. It is generally not a daily necessity but is mainly used to improve the quality of life (Strahilevitz and Myers, 1998; Dhar and Wertenbroch, 2000). Since the CRM campaign is bundled with focus product, the consumer must purchase specific products before making the donation. The researchers have found that the preference for CRM campaigns that bundled with hedonic products was caused by emotional complementarity, in which the altruistic utility provided by CRM campaigns exactly compensated the guilt of hedonic consumption (Strahilevitz and Myers, 1998; Strahilevitz, 1999; Koschate-Fischer et al., 2012). Hence, in the case of buy-one give-one (BOGO), the donation of hedonic products (such as ice cream) to beneficiaries highlights the imprudent nature of products, which leads consumers to believe that their donations do not meet the immediate needs of the beneficiaries, thus reducing their willingness to buy CRM products. On the other hand, the value to beneficiaries of donating practical products such as toothpaste may increase the willingness of consumers to purchase CRM products, as consumers can easily associate with the actual benefits of donating these products to beneficiaries.

In light of the above discussion, we argue that, for practical products, using BOGO instead of BOGM allows consumers to consider the specific functions and practicability of the product, which makes it easier to generate a specific correlation with the use of the product by the recipient, thus generating a higher purchase intention. In contrast, for hedonic products, using BOGM instead of BOGO will increase the consumers’ actual perception that the beneficiaries are being helped so that consumers have a higher willingness to buy CRM products. Taken together, we propose the following hypothesis:

Hypothesis 1. The interaction of the CRM approach and product type affects consumers’ purchase intention. Specifically, for practical products, the purchase intention of BOGO (vs. BOGM) is higher, while for hedonic products, the purchase intention of BOGM (vs. BOGO) is higher.

### The Mediating Role of Perceived Helpfulness

Previous researches have shown that consumers tend to have a good impression of the companies and brands that conduct CRM campaigns (Berger et al., 1999; Chen et al., 2013). For individuals, there is no additional cost to participating in CRM activities except product purchase expense, but additional emotional effects can be achieved. Botti and McGill (2010) noted that consumers view themselves as carrier of CRM activities and perceive a strong causality between their purchasing behavior and the progress and outcome of CRM activities. This causality will inspire consumers to make a more positive evaluation of the results of their actions (Botti and McGill, 2006; Bonini et al., 2010). Furthermore, Robinson et al. (2012) found that the autonomous selection of CRM products can effectively facilitate positive consumer responses by enhancing consumer awareness of individual roles and helpfulness. One of the core evaluation criteria in CRM is how much help could be provided toward beneficiaries (Fisher et al., 2008). According to the sympathy-altruism hypothesis, when consumers consider participating in CRM activities, the perceived helpfulness is considered to be an important factor affecting whether people donate or buy, and their perception of the amount of donations that the recipients can actually receive may be a determining factor (Batson, 1987).

The consumers’ perceived helpfulness of CRM campaigns affects their initial attitudes and behavioral expectations. For practical products, compared with the BOGM, the BOGO is more likely to trigger a specific association between consumers and products and the beneficiaries of CRM, which means that information regarding CRM becomes more persuasive to these consumers and then makes it easier for consumers to access help that beneficiaries actually receive. Moreover, perceived helpfulness will lead to a higher purchase intention of the practical product. While for hedonic products, it can bring pleasure, but it can also result in guilt and vainness (Strahilevitz and Myers, 1998). It is unlike the consumption of practical products that are believed to be necessary to meet basic needs. In this case, BOGM (vs. BOGO) would be better. On account of the perceived helpfulness of giving equal amounts of money instead of hedonic products being higher, adopting BOGM (vs. BOGO) can get a higher purchase intention. Taken together, the following hypothesis is proposed:

Hypothesis 2. Perceived helpfulness of consumers mediates the interaction between CRM approach and product type on purchase intention.

### The Moderating Effect of Information Statement Order

Information statement order refers to the prepositive or post-position of different information. In recent years, many CRM researches discussed the impact of the information framework, including the statement of donation magnitude (Folse et al., 2010; Müller et al., 2014; Yoo et al., 2018) and information content (Grau and Folse, 2007; Bester and Jere, 2012). These researches are mainly based on the information frame effect, which refers to that information upon which receivers make different judgments on the described object; in terms of that information, the narrator adopts different expression methods for the same information (Levin and Gaeth, 1988). Frame effect can be explained by the theory of information processing, which focuses on the cognitive process of individuals facing information with various forms (Peterson et al., 1991). According to this theory, the choice of information receiver is not only affected by individual differences but also by the way of how information is presented and described as well as the background and the order of the information statement. Prior researches on CRM ad mainly focused on evaluating the text representation (Kim and Lee, 2009; Manuel et al., 2014) or visual differences of pictures (Cryder et al., 2017), but the effect of information statement order got little attention. The order of information statement in CRM can be divided into two types: one is to put the product information behind the charity information (product information post-position), and the other is to put the charity information behind the product information (charity information post-position).

The working memory theory proposed by Baddeley and Hitch (1974) can be used to explain the influence of the information statement order and the materiality of the post-position information. Due to the limited capacity of working memory, if the time is limited, people will only consider a certain amount of information, the search speed for relevant information will be relatively faster, and the utilization rate of information will be lower. In this case, individuals tend to consider limited options; consequently, the nearest and hindmost information becomes priority (Baddeley and Hitch, 1974). Furthermore, the belief adjustment model proposed by Hogarth and Einhorn (1992) also explained the effect of information statement order on individual behavior. In this model, people’s belief is a process of anchoring and adjusting. Individuals in the initial state have a belief anchor that is adjusted by subsequent information. After adjustment, people’s belief will produce a new anchoring point, which will lay the foundation for later belief change. The position of the anchoring point and the information before or behind it are important for the change of belief. Therefore, the order of information statement can lead to the final conversion of belief. Hence, we argue that when an individual is faced with a lot of information and needs to make decisions, the postpositive information can have a stronger influence, no matter how complicated the information processing is.

In general, in order to increase the exposure of the product, advertising information often focuses on the product or brand, while charity information plays a relatively minor role. Researches have shown that emphasizing a different content in advertising can lead to different perceptions among consumers (Lafferty and Edmondson, 2009; Samu and Wymer, 2009). We propose that the order of advertising information statement may make consumers have different attitudes toward products through different information points. When charity information is post-position, the product type has a relatively little influence on consumers’ cognition. Whether it is a practical product or a hedonic product, the purchase intention of BOGO is higher than that of BOGM. However, when product information is post-position, for practical products, the purchase intention of BOGO or BOGM does not have a significant difference, while for hedonic products, consumers still prefer BOGM. Taken together, we propose the following hypothesis:

Hypothesis 3. The order of information statement plays a moderating role in the interaction between CRM approach and product type on purchase intention.

The current research aims to explore the interaction between CRM approach and product type on consumers’ purchase intention and to discuss the mediating role of perceived helpfulness in the above-mentioned relationship and whether this impact is contingent on the order of information statement. Experiment 1 studies the main effect and the mechanism of the interaction between CRM approach and product type on consumers’ purchase intention. Experiment 2 studies the moderating effect of information statement order.

## Experiment 1

The purpose of experiment 1 is to test the interaction effect of CRM approach and product type on consumers’ purchase intention and examine the mediating role of perceived helpfulness.

### Pretest

Before the formal experiment, practical products and hedonic products were selected as experimental materials through a pretest. Practical products are mainly used to meet the basic needs of consumers in terms of specific functions and are generally the necessities of daily life. Hedonic products are oriented to experience pleasure and are mainly used to satisfy consumers’ sensory and spiritual pleasure and enjoyment (Strahilevitz and Myers, 1998; Dhar and Wertenbroch, 2000). Through interviews and reference to previous research, the catalog included eight kinds of products: ice cream, shampoo, movie tickets, lamp, toothpaste, sports shoes, chocolate, and toys.

Thirty-seven participants completed the survey and received cash rewards. First, the description of the concept of the product type and product catalog was offered to the participants. After viewing that, the participants were asked to evaluate the practicability, hedonism, and familiarity of the eight products (Okada, 2005; Chang and Yen, 2013). The results showed that toothpaste (*M*_practicability_ = 6.64, *M*_hedonic_ = 2.52) and sports shoes (*M*_practicability_ = 6.87, *M*_hedonic_ = 2.32) were selected as practical products; chocolate (*M*_practical_ = 3.23, *M*_hedonic_ = 6.26) and toy (*M*_practical_ = 3.08, *M*_hedonic_ = 6.47) were selected as hedonic products. Moreover, results of a single-factor ANOVA test also showed that there were significant differences between the four products in their practicability (*F* = 46.10, *p* < 0.001) and hedonism (*F* = 40.53, *p* < 0.001), which indicated that the above-mentioned products could be clearly distinguished. In addition, the four kinds of products were all of high familiarity, and there was no significant difference in familiarity (*F* = 2.06, *p* = 0.11). The above-mentioned results indicated that these materials could be used in further experiments.

### Experimental Design and Subjects

A between-subject design of 2 (CRM approaches: BOGO vs. BOGM) × 2 (product type: practical product vs. hedonic product) was conducted, and the participants were randomly assigned to one of the four groups. We invited 120 participants to participate in the experiment and excluded 12 participants who did not complete the experiment according to the experimental instructions. A total of 108 valid questionnaires were received (male 45.2%, female 54.8%, *M*_age_ = 23.56). Because age, gender, and other demographic information variables did not have a significant influence, the subsequent statistical analysis did not take them into account.

### Experimental Procedures

First, the participants were asked to carefully read the material about a real-life purchase case and imagine themselves as consumers of the situation. The manipulation of product type was based on the pretest; toothpaste represented the practical product and chocolate represented the hedonic product. The manipulation of the CRM approach was based on different scenarios. Specifically, the participants were told that their toothpaste (chocolate) has run out (been eaten) and to be ready to go buy one. There was a product that attracted their attention, and the product attributes satisfied their expectations. At the same time, the brand of this product was doing the following CRM campaign: In order to help poor children in our country who need social care to grow healthier and happier, the brand will donate toothpaste (money equal to the toothpaste) to the poor children with each product sold.

Next, the participants were asked to evaluate purchase intention and perceived helpfulness in turn. The purchase intention was rated by three items adapted from Maheswaran and Meyers-Levy (1990) and Chang and Yen (2013): “I am very likely to buy the product”; “I am very willing to buy the product”; “I would like to recommend the product to others.” Then, the participants reported their perceived helpfulness with the following items: “If you purchase the product, to what extent would you feel that you added value to the cause?”; “If you purchase the product, to what extent would you feel that you helped the cause?”; “If you purchase the product, to what extent would you feel that you contributed to the cause?” (Robinson et al., 2012). The above-mentioned items were all seven-point scales, ranging from totally disagreeing with “1” to completely agreeing with “7.”

Finally, the participants filled up demographic information and the items about product type, which was consistent with pretest as manipulation check (Okada, 2005; Chang and Yen, 2013).

### Results

#### Scale Reliability Test

The purchase intention and perceived helpfulness scales’ Cronbach *α* coefficient was 0.886 and 0.911. The Cronbach *α* coefficients of the above-mentioned scale were all greater than 0.7, which indicated that the scale had high reliability.

#### Manipulation Check

The result suggested that the practicality of toothpaste (*M* = 6.50) was significantly higher than that of chocolate (*M* = 3.65), and the hedonic score of toothpaste (*M* = 2.31) was significantly lower than that of chocolate (*M* = 6.00), indicating that the manipulation of product type was a success.

#### Hypothesis Test of Interaction Effect

**Figure 1:**
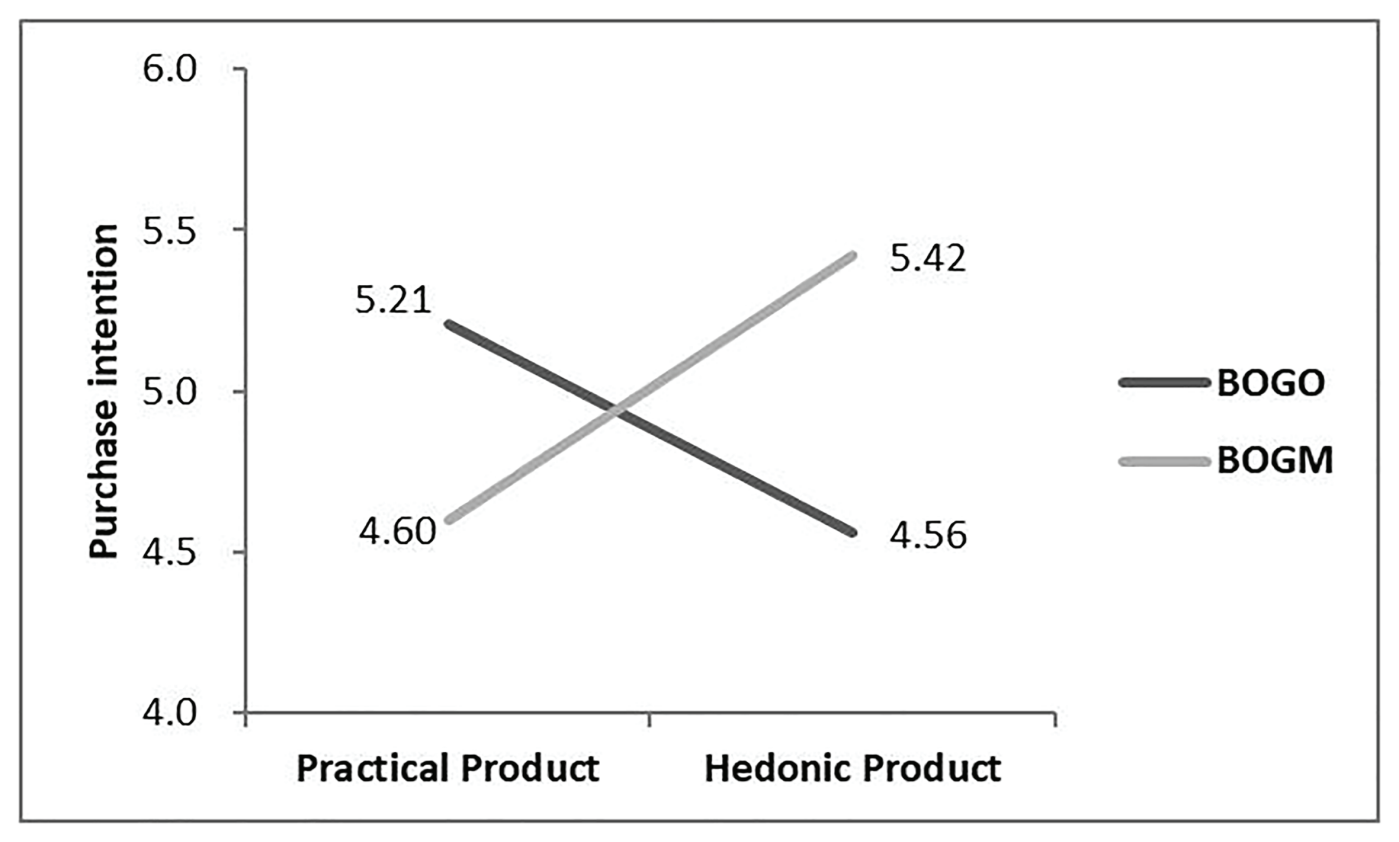
The interaction of cause-related marketing approach and product type on consumer’s purchase intention.

Firstly, an ANOVA with the CRM approach and the product type as independent variables and purchase intention as the dependent variable revealed a significant interaction effect [*F*(1,104) = 8.46, *p* < 0.005, see Figure 1]. The main effect of CRM approach [*F*(1,104) = 0.26, *p* = 0.61] or product type [*F*(1,104) = 0.10, *p* = 0.75] on purchase intention was not significant.

A further analysis showed that, in the practical products condition, the purchase intention of the BOGO group was marginally higher than that of the BOGM group (*p* = 0.09), while for hedonic products, the purchase intention of the BOGM group was significantly higher than the purchase intention when using BOGO (*p* < 0.05). The summarized statistics are reported in Table 2.

**Table 2:** Cause-related marketing approach and product type and purchase intention.

Variables	Practical product		Hedonic product	
	M	SD	M	SD
BOGO	5.21	1.06	4.56	1.13
BOGM	4.60	1.79	5.42	1.14

#### The Mediation Analysis

When considering the mediating role of perceived helpfulness, we referred to the method of Baron and Kenny (1986) for regression analysis. The regression results are presented in Table 3. It revealed that the interaction between CRM approach and product type on purchase intention was significant (*p* < 0.05), and the effect of perceived helpfulness on purchase intention was significant as well (*p* < 0.001). More importantly, the interaction of CRM approach * product type on purchase intention dropped to nonsignificant when we included perceived helpfulness in the model (*p* > 0.05), demonstrating a significant mediating effect. In addition, the result of a bootstrap analysis with 5,000 samples (Hayes, 2013) also showed that the indirect effect of the interaction between CRM approach and product type on purchase intention (95% CI: 0.53, 1.85) did not include zero, confirming that perceived helpfulness fully mediated the effect; these findings provided support for hypothesis 2.

**Table 3:** Regression results – mediating effect of perceived helpfulness.

Variables	Model 1		Model 2		Model 3	
	Purchase intention		Perceived helpfulness		Purchase intention	
	*β*	*P*	*β*	*P*	*β*	*P*
Cause-related marketing approach	0.605	0.093	0.370	0.242	0.379	0.216
Product type	0.654	0.070	1.272	0.000	−0.121	0.711
CRM approach * product type	−1.469	0.004	−1.765	0.000	−0.393	0.394
Perceived helpfulness					0.610	0.000
Adjusted *R*^2^	0.052		0.164		0.319	
Significance	0.037		0.000		0.000	

### Discussion

In the first experiment, we tested the interaction effect of CRM approach and product type on purchase intention and the underlying mechanism – the mediating role of perceived helpfulness – and successfully verified hypothesis 1 and hypothesis 2. The experimental results showed that, for practical products, the CRM approach of BOGO had a higher purchase intention than that of BOGM, while in the hedonic product condition, BOGM had a higher purchase intention than that of BOGO. Moreover, it is preliminarily verified that the interaction effect was mediated by the perception of helpfulness. Next, we will explore which circumstances will strength or weaken the interaction effect between CRM approach and product type on consumers’ purchase intention.

## Experiment 2

The purpose of experiment 2 was to test whether the order of information statement (charity information or product information post-position) of CRM campaigns moderates the interaction between CRM approach and the product type on the purchase intention of this study. We used different products to enhance the robustness of the results.

### Experimental Design and Subjects

Experiment 2 conducted a three-way between-subject design of 2 (CRM approach: BOGO vs. BOGM) × 2 (product type: practical product vs. hedonic product) × 2 (information statement order: charity information post-position vs. product information post-position). We recruited 240 participants to take part in the experiment and excluded 16 participants who did not complete the experiment; 224 valid questionnaires were received (male 40.8%, female 59.2%, *M*_age_ = 23.16).

### Experimental Procedures

In experiment 2, the participants were randomly assigned to one of the eight experimental groups. Firstly, for the manipulation of product type, we chose sports shoes as the practical product and toy as the hedonic product. Specifically, the participants read the description about the purchase scenario and the product introduction. In the practical product condition, the participants were told to imagine that they would like to buy a pair of shoes for the coming Christmas and there was a proper choice when they were shopping in the store. The shoes information was presented in detail, such as price, appearance, and function, while in the hedonic product condition, the participants were told that they want to buy a toy and were presented with the product introduction about a toy, including the price, features, and experience.

In addition, the participants read about the charity information of the CRM campaign that the product was engaged in. For the manipulation of CRM approach, in the BOGO group, the participants were told that if they buy a pair of shoes (a toy) now, the brand would then donate the same product to the Poverty Alleviation Foundation to help the poor children. The participants in the BOGM group were told that if they buy a pair of shoes (a toy) now, the brand would then directly donate money that is equal to the product to the Poverty Alleviation Foundation to help the poor children.

It was worth noting that we presented product information and charity information in different orders, which depended on their group for information statement order manipulation. To be specific, after the participants read the purchase scenario, the charity information post-position group would read charity information after reading product information. On the contrary, the product information post-position group would read the product information after the charity information.

Finally, the participants were asked to evaluate their purchase intention (Maheswaran and Meyers-Levy, 1990; Chang and Yen, 2013) and perceived helpfulness (Robinson et al., 2012) and to report their perceived product attributes as product type manipulation check (Okada, 2005; Chang and Yen, 2013). The questionnaire also measured the demographic variables of the participants. The scales mentioned above were all seven-point scales, and the items were consistent with those of experiment 1.

### Results

#### Manipulation Check

There were significant differences in practicability and hedonism between those two products. The utility score of the sports shoes (*M*_sports shoes_ = 5.84) was significantly higher than that of the toy (*M*_toy_ = 2.39, *t* = 19.25, *p* < 0.001), and the hedonic score of the sports shoes (*M*_sports shoes_ = 3.40) was significantly lower than that of the toy (*M*_toy_ = 5.84, *t* = 18.64, *p* < 0.001). This showed that the product type manipulation was a success.

#### Hypothesis Test of Interaction Effect

**Figure 2:**
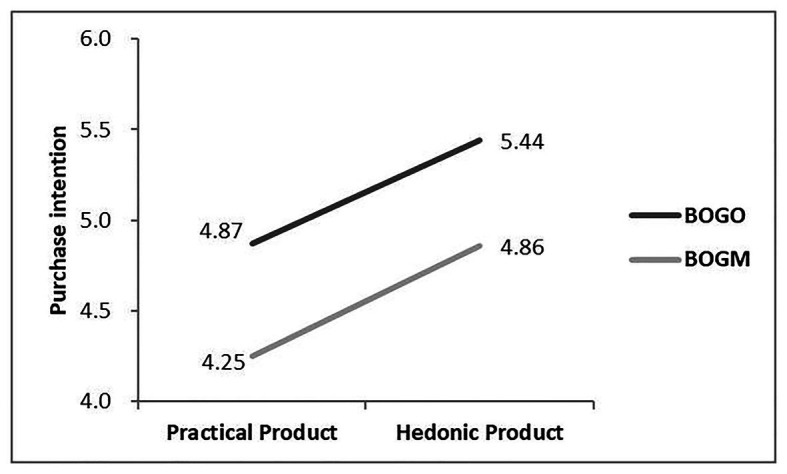
The effect of the interaction of cause-related marketing approach and product type on consumer’s purchase intention when charity information post-position.

**Figure 3:**
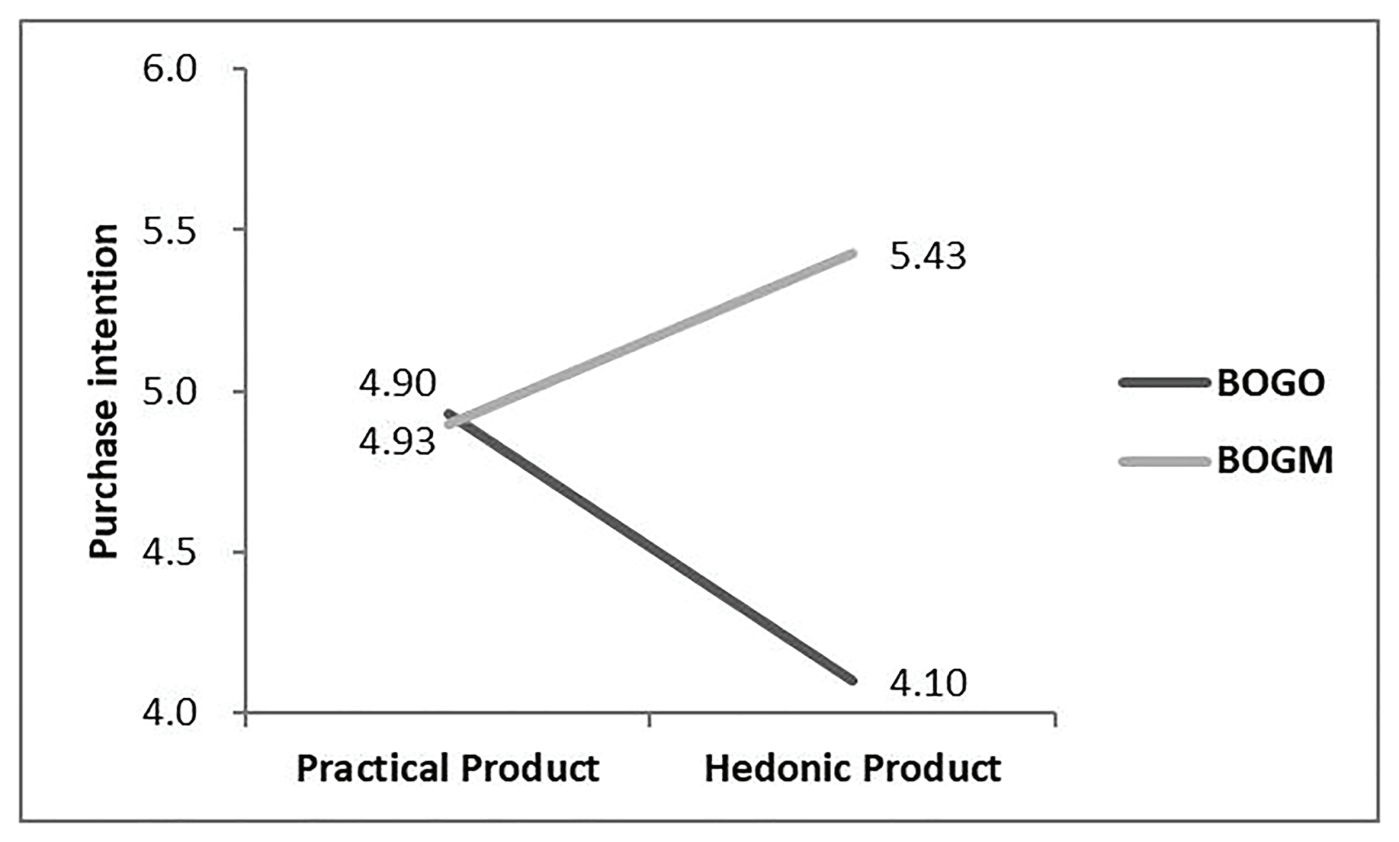
The effect of the interaction of cause-related marketing approach and product type on consumer’s purchase intention when product information post-position.

First, experiment 2 carried a three-way ANOVA. It used CRM approach, product type, and information statement order as independent variables and purchase intention as the dependent variable. The results showed that the main effects of CRM approach [*F*(1,216) = 0.05, *p* = 0.83], product type [*F*(1,216) = 3.14, *p* = 0.08], and information statement order [*F*(1,216) = 0.02, *p* = 0.90] were not significant. All that matters was that the three-way interactive effect of CRM approach, product type, and information statement order was significant [*F*(1,216) = 7.25, *p* = 0.008]. Figures 2, 3 summarize the results.

Then, a simple effect analysis was conducted in terms of the three-way interaction that was significant, and the summarized statistics are reported in Tables 4 and 5. When charity information was post-position, for the practical product, the purchase intention of BOGO was higher than that of BOGM (*p* = 0.012); similarly, for the hedonic product, the purchase intention of BOGO was higher than that of BOGM (*p* = 0.018; see Figure 2). However, when product information was post-position, for the practical product, the purchase intention of BOGO or BOGM did not have a significant difference (*p* = 0.923), while for the hedonic product, the consumers still preferred BOGM (*p* < 0.001, see Figure 3). From these findings, hypothesis 3 was confirmed.

**Table 4:** Cause-related marketing approach and product type and purchase intention when charity information post-position.

Variables	Practical product		Hedonic product	
	M	SD	M	SD
BOGO	4.87	0.87	5.44	0.87
BOGM	4.25	1.12	4.86	0.80

**Table 5:** Cause-related marketing approach and product type and purchase intention when product information post-position.

Variables	Practical product		Hedonic product	
	M	SD	M	SD
BOGO	4.93	0.83	4.10	1.17
BOGM	4.90	0.85	5.43	0.74

#### The Moderated Mediation Analysis

Lastly, according to the mediation analysis program that was proposed by Zhao et al. (2010) and the moderated mediation model (model 9) which was proposed by Preacher et al. (2007), a bootstrap analysis with 5,000 samples was carried out. The results showed that perceived helpfulness did act as a mediator because the direct effect of CRM approach on purchase intention was not significant (95% CI: −0.17, 0.27), but the indirect effect (moderated mediation) was significant (95% CI: 0.09, 0.41), which confirmed hypothesis 2. Specifically, the moderated mediation effect with the order of information statement as the moderating variable was significant (95% CI: 0.09, 0.42), while the moderated mediation effect with product type as the moderating variable (95% CI: −0.06, 0.22) was not significant. From the above-mentioned results, the order of information statement moderated the interaction between CRM approach and product type on the purchase intention, which confirmed hypothesis 3.

### Discussion

Experiment 2 used different products to increase the validity and the applicability of experimental findings. The interaction effect of CRM approach and product type was demonstrated again. Moreover, experiment 2 further discussed the moderated mediation effect and investigated the moderating role that the order of information presentation plays in the influence of charity marketing mode and the product type on purchase intention.

## Conclusion and Discussion

### Conclusion

The current research explores the interaction between CRM approach and product type on the purchase intention. Experiment 1 proved that CRM approach and product type have an interactive effect on purchase intention, and perceived helpfulness plays a mediating role. Specifically, in the practical product condition, BOGO get a higher purchase intention of consumers in terms of the fact that they are more likely to consider the specific product function and practicability and perceive more helpfulness, while for the hedonic product, BOGM will increase the consumers’ perceived helpfulness to the beneficiaries and thus get a higher purchase intention. Furthermore, we argued that the post-position of product information or charity information in an advertisement may have a different impact on consumers’ purchase intention. In experiment 2, we pointed out the moderator role that the information statement order played. When charity information is post-position, whether it is a practical product or a hedonic product, the purchase intention of BOGO is higher than that of BOGM. However, when product information is post-position, for practical products, the purchase intention of BOGO or BOGM does not have a significant difference, while for hedonic products, consumers still prefer BOGM.

### Theoretical Implication

This research provides several theoretical contributions. First, this research enriches the literature on cause-related marketing. Previous studies of CRM have focused on promotional aspects, like cause-brand fit (Trimble and Rifon, 2006; Lafferty, 2007; Lee Thomas et al., 2011; Myers and Kwon, 2013), firm-cause fit (Gupta and Pirsch, 2006; Koschate-Fischer et al., 2012; Elving, 2013), ad type (Hyllegard et al., 2010; Chang, 2011, 2012; Tucker et al., 2012), and ad focus (Lafferty, 2009; Samu and Wymer, 2009), but few studies have focused on CRM approach (BOGO and BOGM) and their respective application scope, not to mention comparing two CRM approaches in one study. Additionally, although there have been some studies on product types in CRM (Strahilevitz and Myers, 1998; Strahilevitz, 1999; Lafferty et al., 2004; Hou et al., 2008; Chang and Liu, 2012), they have not explored the interaction of product type and CRM approach on consumers’ purchase intention. A few researches have previously noted a link between product type and donation, but they have only found that consumers are more likely to donate when they buy a hedonic product (Strahilevitz and Myers, 1998; Strahilevitz, 1999). Based on current research, we demonstrated the conditions under which corporate donation approach may be effective through a simple demonstration of how monetary and non-monetary donations affect the effectiveness of CRM.

Second, this research extends perceived helpfulness into the CRM field. Previous studies have shown that consumers support traditional charity marketing activities in the form of giving money due to sympathy or warm emotions (Strahilevitz, 1999; Pracejus et al., 2003) and have not explored perceived helpfulness of beneficiaries as a mechanism to support the effect of CRM activities. From the definition of helpfulness, we can infer that the more helpful a product is, the more likely it is for the consumer to make a purchase decision. In this study, we conducted empirical experiments to prove that this is the underlying mechanism of the interaction between product types and the CRM approach on purchase intention. Therefore, this study accomplishes empirical research on perceived helpfulness in the field of CRM.

Third, this research extends the information framework from the perspective of information statement orders to the cause-related marketing context and enriches the relevant literature. There have been some studies on the impact of information framework on CRM from the perspective of information content (Grau and Folse, 2007; Bester and Jere, 2012). Specifically, most of existing research on information framework starts from the perspective of simple text or picture form (Kim and Lee, 2009; Manuel et al., 2014; Cryder et al., 2017), and little consideration is given to the influence of the order of information statement. In this study, based on the information processing theory, the interactive effect of product types and cause-related marketing approaches in different information statement orders on consumers’ purchase intention was explored, and a moderating effect on the interaction effect was found. Therefore, it enriches research on information framework.

### Practical Implication

This research can provide practical suggestions for enterprises. Many enterprises claim to be willing to fulfill their social responsibility to devote themselves to public welfare undertakings, but they are deterred from doing so due to concerns about economic benefits and consumers’ uncertain response. According to the main findings of this paper, enterprises can consider the CRM approach for sustainable marketing. Companies planning to support a cause face different donation options (products vs. money), but previous research has not investigated the relative effectiveness of these options. Our results show that, when products are well matched to the way they are donated, consumers would have a higher willingness to buy so that corporate philanthropy can work. To be specific, if the enterprise is mainly engaged in practical product sales (like sports shoes and toothpastes), the way of “buy-one give-one” will make consumers have a higher perception of help and thus generate a greater purchase intention. On the contrary, enterprises engaged in hedonic products sales (like toys and chocolates) still using the way of “buy-one give-one” would not get a higher response from consumers in terms of the product that is for entertainment, and the perceived helpfulness brought by it is marginal. The way of “buy-one give-money” should be considered by enterprises to make consumers have a higher purchase intention since money would bring more perceived helpfulness than hedonic products at this time. Based on our findings, marketers can now design CRM campaigns and make the wisest decisions about how to combine different product types and causes to choose the right corporate donation.

In addition, the results of this study have important practical significance for companies to formulate relevant advertising strategies for public welfare products. Nowadays, consumers can get access to information from various sources through various channels, and the flood of information distracts consumers’ attention. Therefore, how to effectively disseminate information in CRM has been the focus of enterprises gradually. Effective advertising strategies are crucial to promoting consumer response and engagement, especially if the information conveyed in advertisements can be successfully accepted and understood by consumers. The results of our research provide some guidance. First of all, companies should be aware that there are two types of information in advertisements, one is information about the product itself and the other is information about corporate charitable donations. Moreover, the research results showed that, when formulating the charity marketing strategy, enterprises should not only consider which charity mode to choose to cooperate with according to the product but also consider the influence of the order of expression of the above-mentioned two kinds of information on the communication effect. To sum it up, the CRM approach, product type, and information statement order on consumer behavior should all be taken into account when enterprises carry out CRM campaigns.

### Limitation and Future Research Direction

Naturally, this research has limitations which point to directions for future research. Firstly, the effect of high-price products such as luxury has not been explored; we just discussed products of lower prices. Secondly, when it comes to discussing the influence of the interaction between CRM approach and product type on purchase intention, this study just selected the order of information statement as the moderating variable, whereas the relationship from the perspective of consumer characteristics has not been analyzed. This study did not analyze the information processing of how consumers produce attitude and purchase intention as well. Lastly, we have to admit that, due to factors such as time and geographical conditions, our experimental sample group is relatively single; maybe there are limitations in the application and promotion of the results to some extent, and future research should be carried out in a wider range of samples to enhance the external validity of the experiment.

Some new research problems can be derived from this study, with the aim to provide a certain expansion direction for future research. First, there are many different classification methods for the degree of conformity in CRM. Although some research have focused on the impact of the degree of compatibility between business and case on consumers’ psychology and behavior, other classification methods could be chosen for future research, such as resource fit and employee fit. Second, advertising is a complicated way of propaganda; besides the expression of words in CRM advertisement, there are many other factors that may influence the consumer’s attitude toward CRM, such as the composition and hue of the picture, which can be verified in future studies. Finally, different consumers have different responses to CRM. Later research can take the characteristics of consumers into account, such as the consumers’ moral identity.

## Data Availability Statement

The original contributions presented in the study are included in the article/Supplementary Material, further inquiries can be directed to the corresponding author.

## Ethics Statement

The studies involving human participants were reviewed and approved by the ethics committee of the School of Management, Jinan University, China. All participants gave their written informed consent before the experiments.

## Author Contributions

SY, YL, and HC conceived and designed the experiments, and carried out the experiments. YL analyzed the experimental results. SY, YL, and SG wrote the manuscript. SG edited the manuscript. All authors contributed to the article and approved the submitted version.

### Conflict of Interest

The authors declare that the research was conducted in the absence of any commercial or financial relationships that could be construed as a potential conflict of interest.
